# Soybean Crop Response Treated With Different Multifunctional Inoculants Under Field Conditions

**DOI:** 10.1111/1758-2229.70363

**Published:** 2026-05-15

**Authors:** Cássia Cristina Rezende Mirza, Ana Paula Santos Oliveira, Maria Eduarda Silvério Mateus, Princewill Chukwuma Asobia, Cleiton Mateus Sousa, Laylla Luanna de Mello Frasca, Mariana Aguiar Silva, Enderson Petrônio de Brito Ferreira

**Affiliations:** ^1^ Universidade Federal de Goiás Goiânia Goiás Brazil; ^2^ Instituto Federal Goiano—Campus Ceres Ceres Goiás Brazil; ^3^ Embrapa Arroz e Feijão Santo Antônio de Goiás Goiás Brazil

**Keywords:** bioinputs, *Glycine max*, grain yield, phytohormones, rhizobacteria

## Abstract

Soybean is the main crop in Brazilian agribusiness, but its production is limited by low soil phosphorus availability. An alternative to mitigate this constraint is the use of plant growth‐promoting bacteria capable of solubilising phosphorus and producing phytohormones. This study aimed to evaluate soybean performance in response to different multifunctional inoculants under field conditions. Experiments were carried out in the 2021/22 and 2022/23 seasons in Santo Antônio de Goiás and in 2021/22 in Ceres, Brazil. The experimental design was a randomized block with 20 treatments and five replications. Treatments consisted of seed inoculation of DonMario 68i69 IPRO and NS 6906 IPRO cultivars with phosphate‐solubilising bacteria (BRM 063573, BRM 67205, BRM 67207), auxin‐producing bacteria (BRM 063574, BRM 36549, BRM 67206) and their pairwise combinations, plus five controls (Ab‐V5, BiomaPhos, T0, T50, T100). Data were analysed using multivariate analysis in R and variance analysis (*F* test) at a 5% significance level, followed by Scott‐Knott test. The combination BRM 67207 ( *Bacillus subtilis* ) + BRM 67206 ( *Bacillus pumilus* ) showed the best results across all experiments, enhancing pods per plant, grain yield, root biomass, total root length and root volume. This combination stands out as a promising bioinput for soybean production.

## Graphical abstract

Consortia of plant growth‐promoting rhizobacteria (PGPR) enhance soybean phosphorus use efficiency and grain yield under contrasting field conditions. These bioinputs reduce dependence on mineral fertilizers and support eco‐efficient agriculture, offering a sustainable alternative in diverse soil and climate conditions.

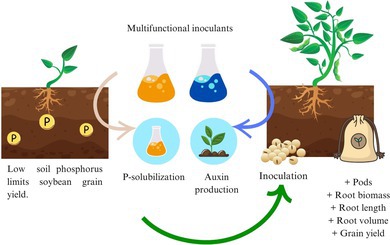

## 1 Introduction

Soybeans are of great importance on the world stage, requiring high investments in research aimed at increasing grain yield (GY) without a significant increase in production costs aimed at maintaining or using new production areas (Chagas Junior et al. 2022). However, despite Brazil's favourable climate and currently being the world's largest soybean producer and exporter, Brazilian production is often affected by nutritional problems, mainly related to phosphorus (Pavinato et al. 2020).

Phosphorus (P) is a macronutrient obtained from phosphate rock, a finite and non‐renewable resource. Its impact on the soil can lead to a reduction in crop yields of up to 15% (Elhaissoufi et al. 2021), because as well as damaging respiration and photosynthetic processes, it leads to flower abortion and reduced grain quality (Bargaz et al. 2021). A recent proposal to address this challenge is the multifunctionality of biological products. Combinations of bacterial strains and even combinations of different microorganisms with multiple functions have been used to extend the range of action of these products (Oliveira‐Paiva 2022).

Recent studies have demonstrated the potential of plant growth‐promoting bacteria (PGPB), used either individually or in combination, to enhance crop growth, nutrient acquisition and yield. Experimental evidence indicates that bacterial consortia may outperform single‐strain inoculants by integrating complementary mechanisms, such as phosphorus solubilization, biological nitrogen fixation, phytohormone production and stimulation of root system architecture, resulting in improved nutrient uptake and plant performance (Bakhshandeh et al. 2020; Elhaissoufi et al. 2021; Oliveira‐Paiva 2022). Despite these promising results, most studies have been conducted under controlled or greenhouse conditions or have focused on isolated mechanisms of action. Field‐scale evaluations of multifunctional inoculants that simultaneously enhance phosphorus use efficiency (PUE) and plant growth in soybean remain limited, particularly under Brazilian production conditions.

In this sense, the PGPBs use represents a safe and environmentally friendly alternative in agricultural production systems, promoting the sustainability of agroecosystems (Dias and Santos 2022). PGPBs are capable of stimulating plant growth at different stages of development through direct mechanisms, such as phosphorus acquisition, nitrogen fixation and even modulation of phytohormone levels, leading to increased root surface area and better vegetative growth (Bakhshandeh et al. 2020; Sousa et al. 2021).

The nitrogen (N) fixing inoculants use is already a reality in the soybean production chain and phosphate solubilising inoculants and growth regulators have gained prominence in recent decades, but there is currently no multifunctional commercial inoculant in Brazil that combines these two mechanisms of action (Oliveira‐Paiva 2022). In this sense, it is important to carry out studies that assess the ability of these microorganisms to make P available from the soil as a more sustainable alternative for using the nutrient. The aim of this study was to evaluate the performance of the soybean crop in response to different multifunctional inoculants under field conditions.

## 2 Material and Methods

### 2.1 Environmental Characterization

**FIGURE 1:**
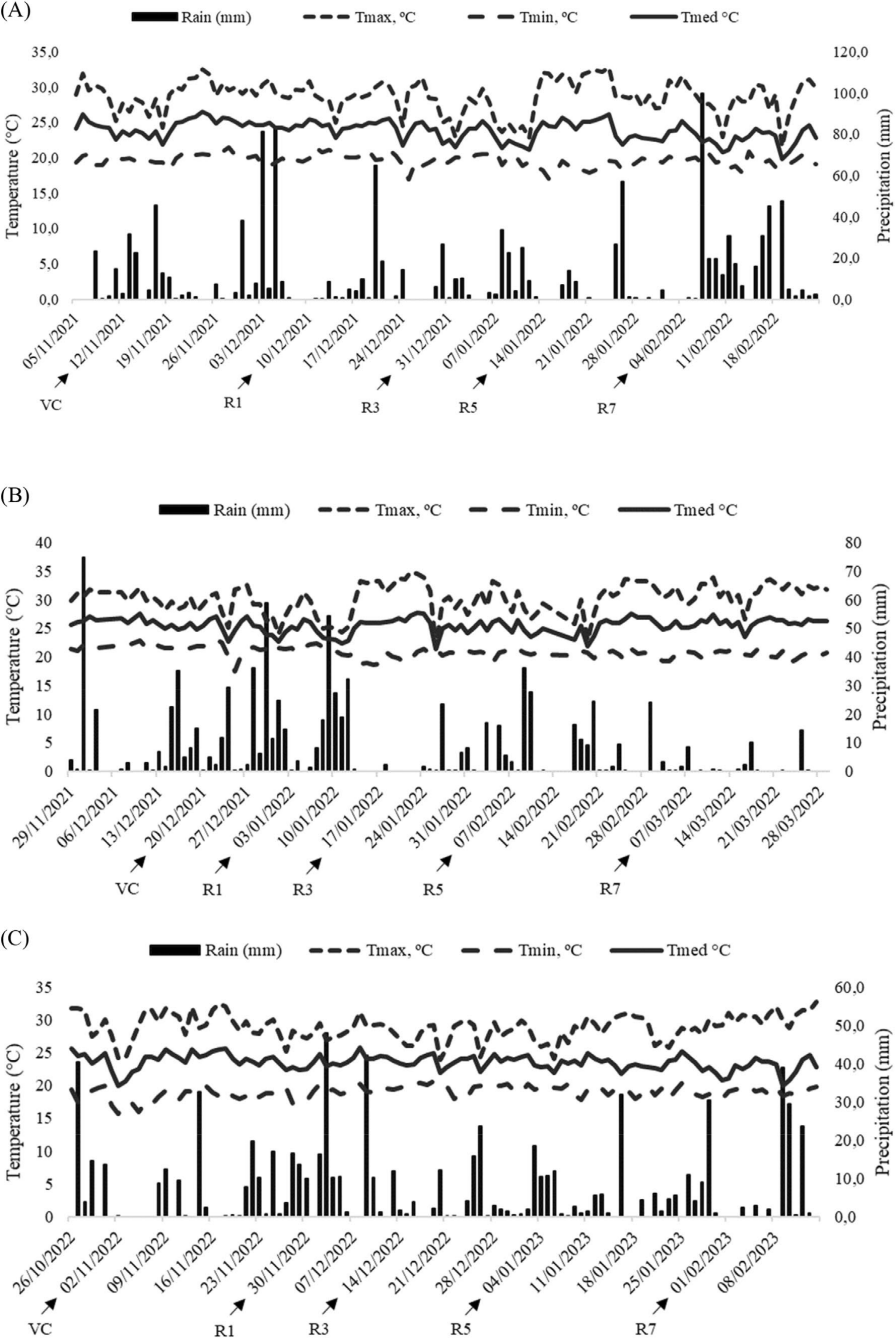
Temperature and rainfall during the soybean cycle in the 2021/2022 harvest in (A) Santo Antônio de Goiás, GO (B) Ceres, GO and in the 2022/23 harvest in (C) Santo Antônio de Goiás, GO, showing the phenological phases of the crop: VC = cotyledon; R1 = flowering start; R3 = pod formation start; R5 = grain filling start; R7 = ripening start.

Three experiments were conducted under field conditions, two in the 2021/22 and 2022/23 harvests at the Experimental Station of Embrapa Arroz e Feijão, located in Santo Antônio de Goiás, GO, Brazil (latitude 16°29′13.90″ S, longitude 49°17′47.68″ W and altitude 767 m); and another in the 2021/22 harvest at the Instituto Federal Goiano—Campus Ceres, located in Ceres, GO, Brazil (latitude 15°21′18.65″ S, longitude 49°36′26.26″ W). According to the Köppen classification (Köppen and Geiger 1936), the two locations climate is tropical savannah Aw (tropical with wet summer and dry winter). During the harvest, temperature and rainfall data were recorded for both locations (Figure 1A–C).

Before setting up the experiments, the soil was chemically analysed according to the methodology proposed by Melo et al. (2017) (Table 1).

**TABLE 1:** Soil chemical attributes of the experimental areas in Santo Antônio de Goiás—GO and Ceres—GO in the 2021/22 harvest.

Location/harvest	pH	O.M.	P			K	Ca			V%
		%	mg dm^−3^	Al	H + Al	cmol_c_ dm^−3^		Mg	CEC	%
SAG*—2021/22	5.1	2.5	14.2	0.0	3.0	0.2	2.5	1.1	6.8	55.2
CE—2021/22	5.2	1.2	18.3	0.1	2.2	0.2	2.4	1.4	6.2	64.5

Abbreviations: CE = Ceres—GO; SAG = Santo Antônio de Goiás—GO.

* Embrapa Arroz e Feijão uses a chemical analysis for two consecutive years, so a new analysis was not carried out for the 2022/23 harvest.

### 2.2 Experimental Design and Treatments

A randomized block design was used, with 20 treatments and five replicates. The treatments consisted of inoculating soybean seeds with different bacteria and their combinations in pairs; three phosphate‐solubilising isolates bacteria (BRM 063573, BRM 67205 and BRM 67207), three indole‐3‐acetic acid (IAA)‐producing isolates bacteria (BRM 063574, BRM 36549 and BRM 67206), as well as five control treatments (Ab‐V5, BiomaPhos, T0—no inoculation and P fertilization, T50—no inoculation and 50% P fertilization and T100—no inoculation and 100% P fertilization), making a total of 20 treatments. The bacteria used are stored and preserved in the Multifunctional Microorganisms collection at Embrapa Arroz e Feijão.

The treatments and the main characteristics of the bacterial isolates are described in Table 2.

**TABLE 2:** Treatments complementary to inoculation with *Bradyrhizobiun japonicum* before planting with growth‐promoting bacteria and chemical fertilization with Phosphorus in the soybean crop.

Treatments^a^	Inoculation^b^	P solubilizer^c,d^	IAA producer^c,d^	Commercial inoculant^e^	P fertilization^f^
1 (1381)	+	−	BRM 063574 ( *Stenotrophomonas maltophilia* )	−	−
2 (Ab‐V6)	+	−	BRM 36549 ( *Azospirillum brasilense* )	−	−
3 (1341)	+	−	BRM 67206 ( *Bacillus pumilus* )	−	−
4 (1301)	+	BRM 63573 ( *Bacillus pumilus* )	−	−	−
5 (1301 + 1381)	+	BRM 63573	BRM 063574	−	−
6 (1301 + Ab‐V6)	+	BRM 63573	BRM 36549	−	−
7 (1301 + 1341)	+	BRM 63573	BRM 67206	−	−
8 (1254)	+	BRM 67205 ( *Paenibacillus pabuli* )	−	−	−
9 (1254 + 1381)	+	BRM 67205	BRM 063574	−	−
10 (1254 + Ab‐V6)	+	BRM 67205	BRM 36549	−	−
11 (1254 + 1341)	+	BRM 67205	BRM 67206	−	−
12 (S22)	+	BRM 67207 ( *Bacillus subtilis* )	−	−	−
13 (S22 + 1381)	+	BRM 67207	BRM 063574	−	−
14 (S22 + Ab‐V6)	+	BRM 67207	BRM 36549	−	−
15 (S22 + 1341)	+	BRM 67207	BRM 67206	−	−
16 (TA)	−	−	−	Ab‐V5	−
17 (TB)	−	−	−	BiomaPhos	−
18 (T0)	−	−	−	−	0%
19 (T50)	−	−	−	−	50%
20 (T100)	−	−	−	−	100%

*Note:* ^a^Treatments and numerical code of the plant growth‐promoting isolates bacteria in the Multifunctional Microorganisms collection at Embrapa Arroz e Feijão; ^b^Treatments with plant growth‐promoting bacteria inoculation; ^c,d^Taxonomic classification and biochemical characterization of the isolate (phosphatase or indole‐3‐acetic acid (IAA) producer); ^e^Treatments with commercial inoculants; ^f^Treatments without/with phosphate fertilization; Sequencing reference for each isolate: Asobia (2023), Hungria et al. (2018), and Braga et al. (2018).

### 2.3 Seed Inoculation

Before sowing, the soybean seeds were inoculated with *Bradyrhizobiun japonicum*. The seeds were then treated with the bacteria established for each treatment (with the exception of treatments T0, T50 and T100), all at a suspension concentration of 1 × 10^8^ colony‐forming units (CFU) per mL, following the recommendation of 300 mL ha^−1^. The inoculation process was carried out in a shaded environment, around 2 h before sowing.

### 2.4 Planting the Crop

The 2021/22 crop was planted in November, while the 2022/23 crop was planted in October. The DonMario 68I69 IPRO cultivar was used in the 2021/22 harvest and the NS 6906 IPRO in the 2022/23 harvest. They were mechanically sown at a spacing of 0.5 m between rows, distributing 15 seeds per linear meter. The plots were made up of six rows measuring 3 × 5 m, totalling 15 m^2^.

Fertilization at sowing was carried out according to the soil analysis and the experimental design. The recommended fertilization in Santo Antônio de Goiás in the 2021/22 and 2022/23 harvests was 200 kg/ha, using MAP (monoammonium phosphate) in the 2021/22 harvest and the 0–20–20 formulation in the 2022/23 harvest. In Ceres in the 2021/22 harvest, the recommended fertilization was 100 kg/ha, using MAP. The treatments with microorganisms received a standard fertilization (50% P fertilization recommended after soil analysis). Treatment T100 received 100% of the recommended P fertilization, while T0 received no P fertilization. Cultivation practices and irrigation were carried out according to the recommendations and needs of the crop.

### 2.5 Evaluations

#### 2.5.1 Biomass Production

The shoot part and roots of three plants were collected (39 DAS in SAG 2021/22, 37 DAS in ce 2021/22 and 48 in SAG 2022/23), packed and dried in a forced ventilation oven at 65°C until constant mass for biomass determination.

#### 2.5.2 Root System Characteristics

Before drying, the roots were photographed with a digital camera and the images processed in WinRHIZO 2012 software, Regent Instruments Inc., Quebec City, QC, Canada (Arsenault et al. 1995), analysing: total root length (TRL) (LengR, cm), total root surface area (TRSA) (AreaSR, cm^2^), root diameter (RD) (DiamR, mm) and root volume (RV) (VolR, cm^3^).

#### 2.5.3 Grain Yield and Production Components

The soybean plants were harvested when they reached physiological maturity (112 DAS in SAG 2021/22, 122 DAS in ce 2021/22 and 128 DAS in SAG 2022/23). Ten plants were collected at random from the useful area of each plot to determine the pods number (PN) per plant and the grains number per plant. GY was determined by mechanically collecting all the plants in the central area of each plot to estimate grain weight in kg ha^−1^, with the data corrected to 13% moisture.

### 2.6 Statistical Analysis

The data was submitted to multivariate analysis using R software, where principal component analysis (PCA) was carried out to describe the correlation between the treatments and the response variables and cluster analysis (dendrogram) to explore the similarity between the treatments. The GY data was submitted to variance analysis (*F* test) and, when significant differences were observed between the treatments, the means were compared using the Scott‐Knott test at a 5% error probability.

## 3 Results

Soybean growth traits and yield components were influenced by PGPB and their specific combinations. The effectiveness of each rhizobacterial consortium varied according to soil and climatic conditions, highlighting the environmental specificity of microbial interactions.

The correlation coefficients among the variables differed across experiments, with the most relevant correlations exceeding 0.4. Correlations with the same sign indicated positive associations between the variables. In the PCA, TRL consistently emerged as the most significant variable in PCA 1 across all experiments, while root dry mass (RDM) was the most prominent in PCA 2. Yield components, pods number and grains number (GN), varied in their contributions to the principal components: they were more influential in PCA 1 for the Ceres 2021/2022 season and in PCA 2 for the Santo Antônio de Goiás 2022/2023 season. Additionally, total phosphorus (TP) and PUE were particularly relevant in the Santo Antônio de Goiás site across both seasons (Table 3).

**TABLE 3:** Correlation coefficients between the evaluated variables and the first two principal components (PCA 1 and PCA 2) for experiments conducted in two locations—Ceres (CE) and Santo Antônio de Goiás (SAG)—across three growing seasons (2021/22 and 2022/23).

Variables	SAG 21/22		ce 21/22		SAG 22/23	
	PCA 1	PCA 2	PCA 1	PCA 2	PCA 1	PCA 2
SDM	−0.280	0.329	0.059	−0.345	0.129	0.382
RDM	−0.245	0.454	0.155	−0.398	0.121	0.413
PN	−0.294	−0.285	0.448	0.423	−0.207	−0.439
GN	−0.289	−0.006	0.383	0.465	0.121	−0.500
GY	0.224	−0.109	−0.119	−0.212	0.152	0.189
TRL	−0.307	0.239	0.385	−0.401	0.412	−0.161
TRSA	−0.399	−0.001	0.470	−0.265	0.043	−0.156
RD	−0.094	−0.092	−0.338	0.195	−0.338	0.259
RV	−0.489	0.124	0.140	0.100	0.208	−0.283
TP	−0.264	−0.501	0.177	0.028	−0.528	−0.053
PUE	0.266	0.511	−0.273	−0.054	0.529	0.062

Abbreviations: GN = grains number; GY = grain yield; PN = pods number; PUE = phosphorus use efficiency; RD = root diameter; RDM = root dry mass; RV = root volume; SDM = shoot dry mass; TP = total phosphorus; TRL = total root length; TRSA = total root surface area.

### 3.1 Santo Antônio de Goiás—2021/22

PCA identified two principal components with eigenvalues greater than 1, explaining 48.8% of the total variance in the dataset. Although the cumulative variance explained by PCA 1 and PCA 2 was moderate, the separation of variables and treatments along these components revealed biologically meaningful and agronomically relevant patterns. This distribution of variance is expected in multifactorial agronomic systems, where yield, nutrient use efficiency and plant growth traits respond differently to management practices and environmental interactions.

**FIGURE 2:**
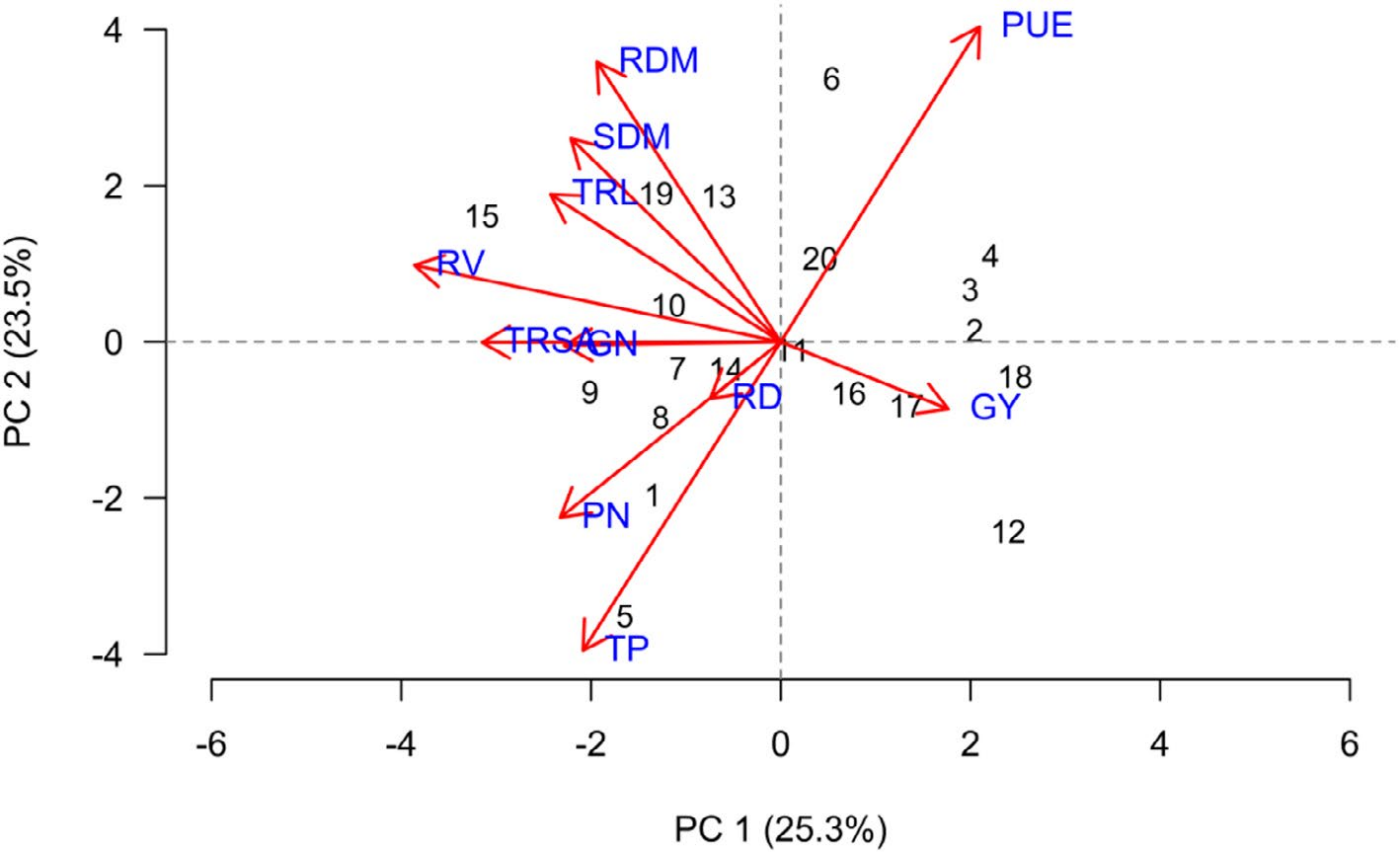
Principal component analysis (PCA) showing correlations between variables and treatments: (1) 1381; (2) Ab‐V6; (3) 1341; (4) 1301; (5) 1301 + 1381; (6) 1301 + Ab‐V6; (7) 1301 + 1341; (8) 1254; (9) 1254 + 1381; (10) 1254 + Ab‐V6; (11) 1254 + 1341; (12) S22; (13) S22 + 1381; (14) S22 + Ab‐V6; (15) S22 + 1341; (16) TA; (17) TB; (18) T0; (19) T50; (20) T100—for soybean grown in Santo Antônio de Goiás in the 2021/22 season.

PCA 1 accounted for 25.3% of the total variation and was predominantly driven by PUE and GY, which exhibited a strong positive correlation. This indicates that PCA 1 represents a productivity–nutrient use efficiency axis, in which treatments positioned further to the right of the ordination plot tend to present higher GY associated with improved PUE. In this context, treatments 2, 3, 4 and 18 showed a clear association with PCA 1, highlighting their superior agronomic performance under the evaluated conditions (Figure 2).

PCA 2 was mainly composed of variables related to plant biomass allocation and root system development, particularly RDM, SDM and TRL, which showed positive correlations among themselves. This component reflects a growth and root architecture axis, indicating that variation along PCA 2 is primarily associated with differences in biomass accumulation and root development rather than yield directly. Treatments 10, 13 and 19 were more strongly associated with PCA 2, suggesting that these treatments favoured vegetative growth and root system (Figure 2).

**FIGURE 3:**
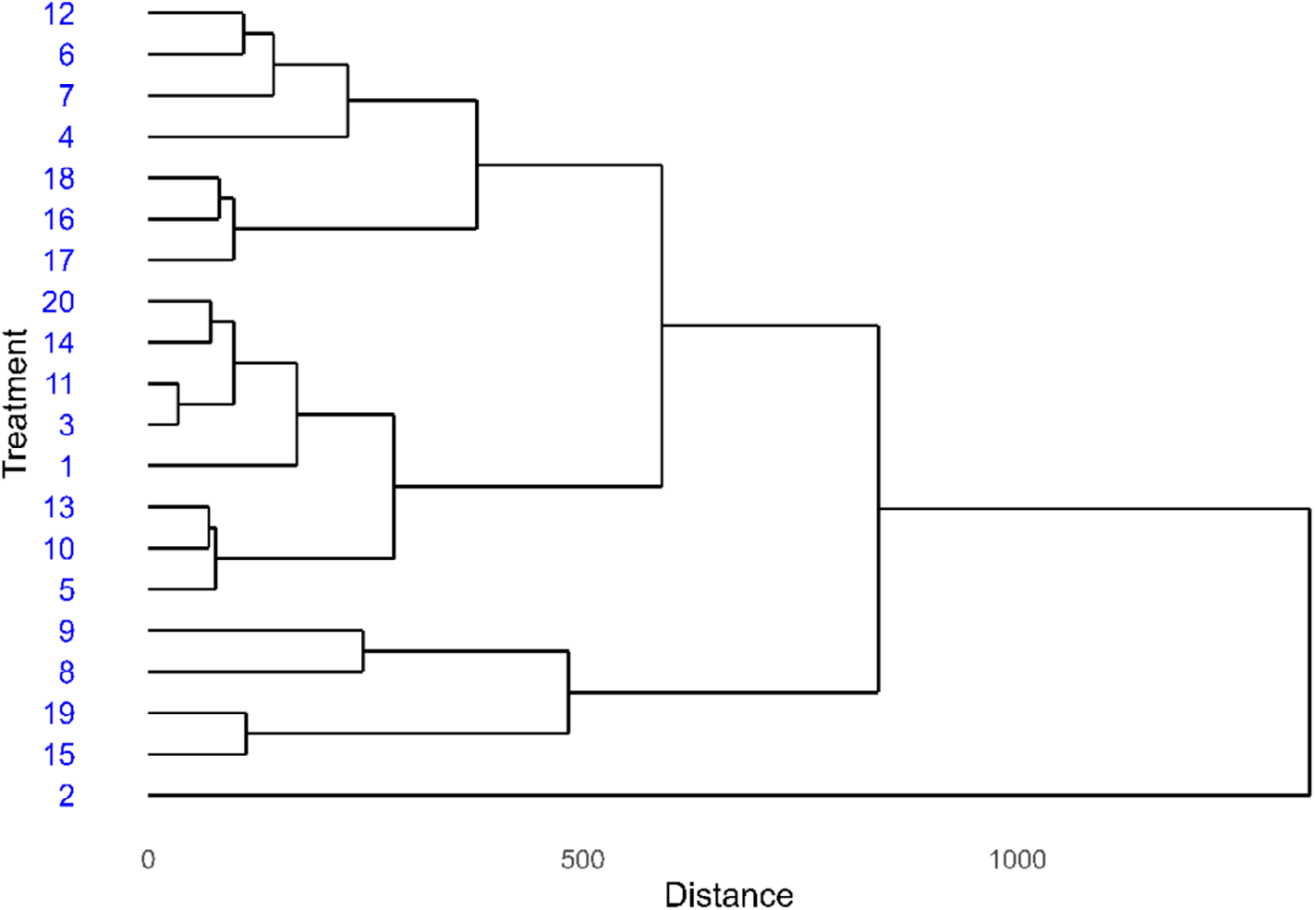
Cluster analysis (dendrogram) showing the similarity among soybean treatments in Santo Antônio de Goiás during the 2021/22 season.

The 20 treatments were grouped into three clusters based on the evaluated variables. Group 1 included treatments 12, 6, 7, 4, 18, 16 and 17, forming a cohesive group that indicates similar responses to the studied variables. Group 2 consisted of treatments 20, 14, 11, 3, 1, 13, 10 and 5, showing a slightly wider variation among them. Group 3 included treatments 9, 8, 19, 15 and 2, which were more distinct in their responses (Figure 3).

Treatment 2 appeared isolated in the dendrogram and was positioned near GY and PUE in the PCA plot, indicating a uniquely positive performance. In contrast, treatments 12, 6 and 7, though grouped together in the dendrogram, were located farther from the GY vectors in the PCA, reinforcing that these treatments contributed less to yield‐related parameters (Figure 3).

### 3.2 Ceres—2021/22

In Ceres during the 2021/22 growing season, PCA identified two principal components (PCA 1 and PCA 2) with eigenvalues greater than 1, jointly explaining 39.4% of the total variance. Although this cumulative contribution is moderate, it reflects the inherent complexity of agronomic systems, in which multiple traits contribute to plant performance and GY.

**FIGURE 4:**
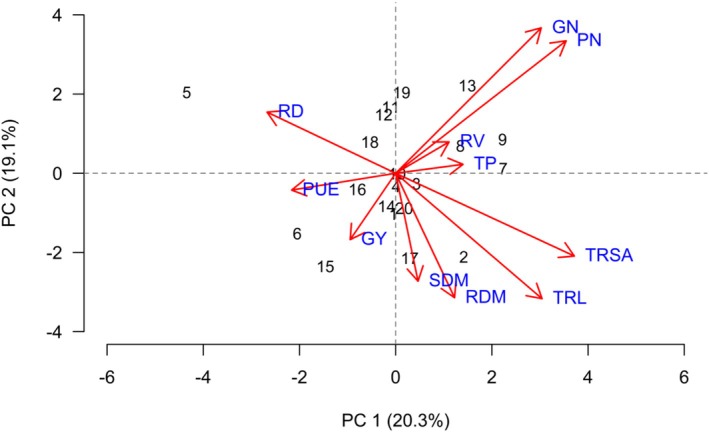
Principal component analysis (PCA) showing correlations between traits and treatments: (1) 1381; (2) Ab‐V6; (3) 1341; (4) 1301; (5) 1301 + 1381; (6) 1301 + Ab‐V6; (7) 1301 + 1341; (8) 1254; (9) 1254 + 1381; (10) 1254 + Ab‐V6; (11) 1254 + 1341; (12) S22; (13) S22 + 1381; (14) S22 + Ab‐V6; (15) S22 + 1341; (16) TA; (17) TB; (18) T0; (19) T50; (20) T100 for soybeans cultivated in Ceres during the 2021/22 season.

PCA 1 was predominantly associated with variables related to plant biomass accumulation and root system development. TRL, TRSA, SDM and RDM exhibited strong positive correlations and were mainly influenced by treatments 2 and 17, indicating enhanced vegetative growth and root development under these treatments (Figure 4). These traits collectively define a growth and root architecture axis, highlighting the importance of below‐ and aboveground biomass allocation in treatment differentiation.

The production components pods number and grains number (GN) were strongly and positively correlated with each other; however, they were not directly associated with GY, suggesting that yield variation in this environment was influenced more by physiological and root‐related traits than by yield components alone. Notably, treatment 2 stood out for its strong association with the root and shoot development variables (SDM, RDM, TRL and TRSA) and was positioned close to the GY vector, indicating a potential contribution of enhanced root development to increased GY (Figure 4).

In contrast, treatments 5, 6 and 15 were located in the negative quadrant of PCA 1 and were associated with RD, PUE and GY, suggesting a distinct agronomic response pattern characterized by differences in nutrient use efficiency and root morphology. Treatments 7, 8 and 9 were positioned near the vectors of RV and TP, indicating that these variables played a more prominent role in their agronomic performance. The remaining treatments showed intermediate behaviour and clustered near the centre of the biplot, reflecting a balanced or less pronounced response across the evaluated traits (Figure 4).

**FIGURE 5:**
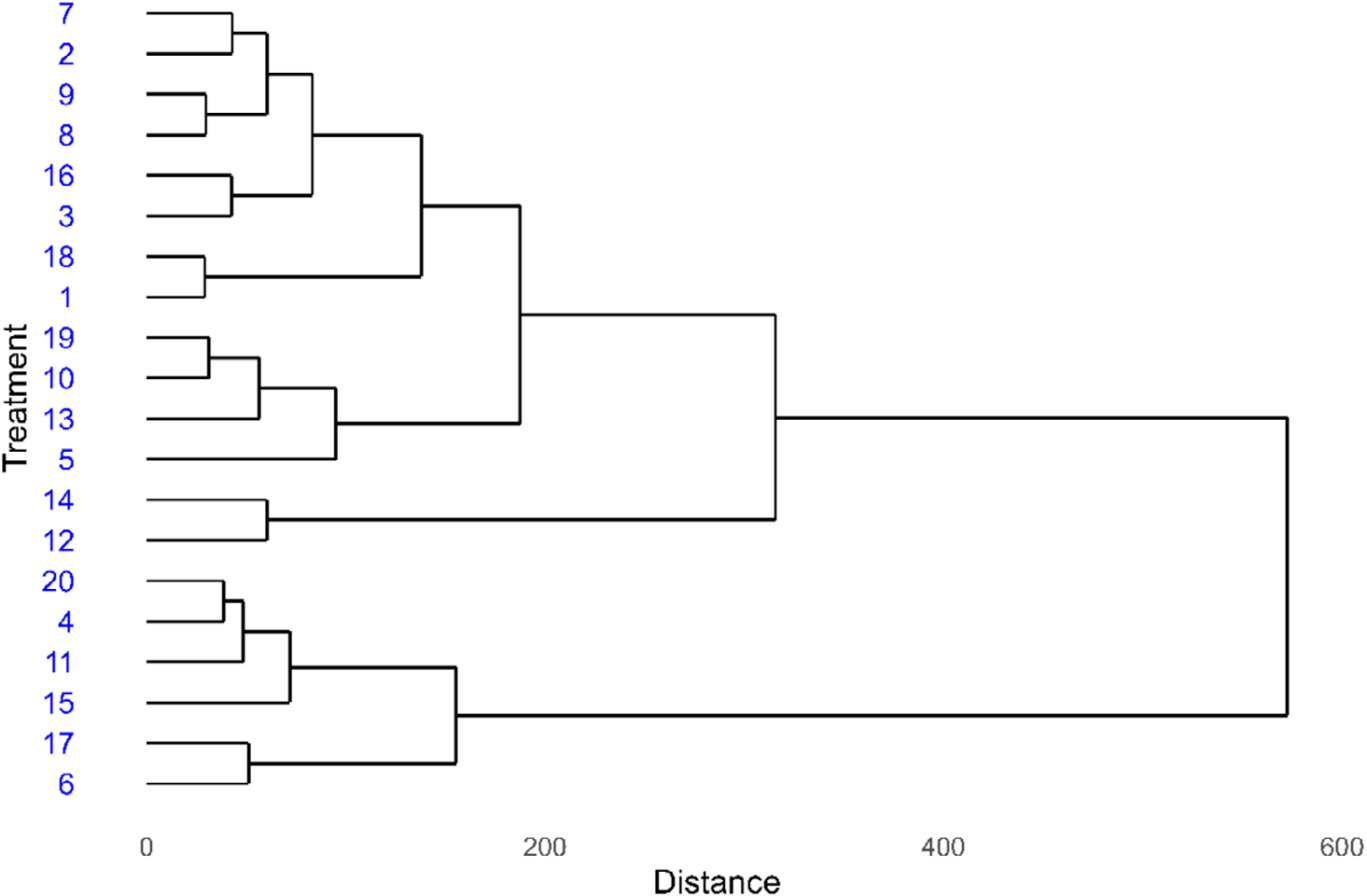
Cluster analysis (dendrogram) showing similarity among soybean treatments in Ceres during the 2021/22 season.

The dendrogram based on the soybean treatments applied in Ceres during the 2021/22 season revealed the formation of four distinct groups. Treatments 7, 2, 9, 8, 16, 3, 18 and 1 formed the most cohesive group, indicating highly similar responses under the experimental conditions. This similarity suggests that the inoculants used in these treatments had comparable effects on the analysed traits (Figure 5).

Another group comprised treatments 19, 10, 13 and 5, also indicating a similar agronomic pattern. Treatments 14 and 12 formed a third group, reflecting comparable performance of the microorganisms involved. The fourth group, consisting of treatments 20, 4, 11, 15, 17 and 6, was more distinct, indicating divergent behaviour likely due to specific interactions between microbial strains and environmental conditions (Figure 5).

### 3.3 Santo Antônio de Goiás—2022/23

The principal components PCA 1 and PCA 2 presented eigenvalues greater than 1 and jointly explained 51.0% of the total variance in the soybean dataset. This cumulative contribution indicates that a substantial portion of the variability was captured by the first two components, allowing a robust interpretation of the relationships among variables and treatments.

**FIGURE 6:**
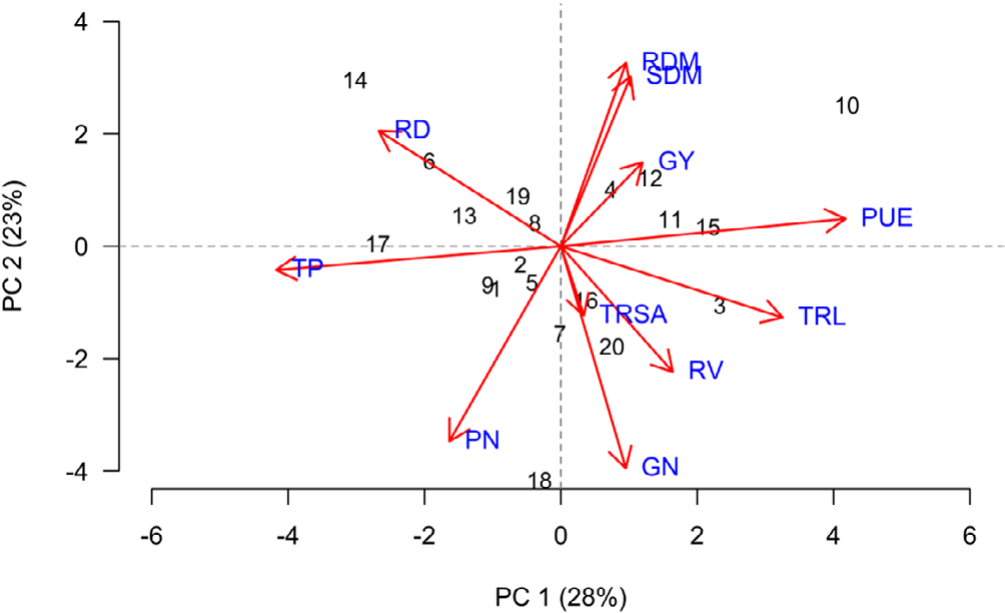
Principal component analysis (PCA) showing correlations between variables and treatments: (1) 1381; (2) Ab‐V6; (3) 1341; (4) 1301; (5) 1301 + 1381; (6) 1301 + Ab‐V6; (7) 1301 + 1341; (8) 1254; (9) 1254 + 1381; (10) 1254 + Ab‐V6; (11) 1254 + 1341; (12) S22; (13) S22 + 1381; (14) S22 + Ab‐V6; (15) S22 + 1341; (16) TA; (17) TB; (18) T0; (19) T50; (20) T100 for soybeans cultivated in Santo Antônio de Goiás during the 2022/23 season.

PCA 1 was predominantly defined by variables related to nutrient use efficiency, biomass accumulation and yield‐related traits. PUE, TRL, grains number (GN), SDM and RDM showed strong positive correlations and were positioned in the positive quadrant of this axis. These variables were mainly responsible for the separation of treatments located on the right side of the biplot, including treatments 3, 4, 10, 11, 12, 15, 16 and 20, indicating superior agronomic performance and positive differentiation relative to the other treatments (Figure 6).

Among these, treatments 4 and 12 showed the strongest association with GY, suggesting a direct contribution of improved nutrient use efficiency and biomass accumulation to GY. Treatment 16 was more closely associated with TRSA, while treatment 3 showed a strong contribution to TRL, highlighting treatment‐specific strategies for root system development.

In contrast, RD and TP in the shoot showed little or no correlation with production‐related variables, indicating that increases in these traits were not directly translated into higher GY. Consequently, treatments 14 and 17 were positioned away from the GY axis, suggesting that these treatments may have promoted root thickening and phosphorus accumulation in the shoot without corresponding gains in yield (Figure 6).

**FIGURE 7:**
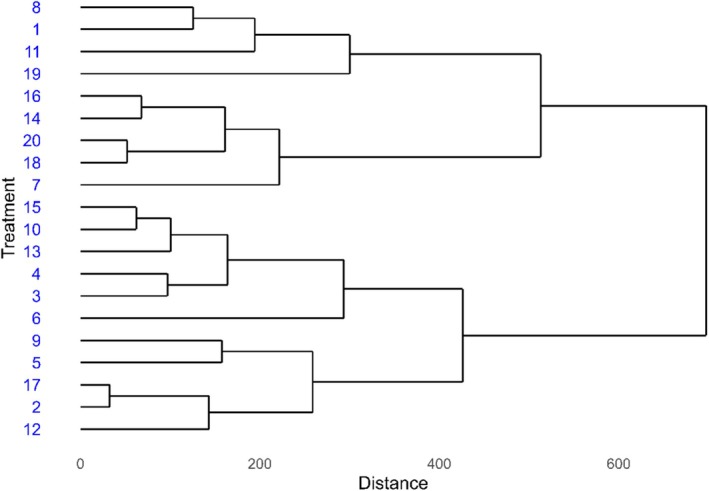
Cluster analysis (dendrogram) showing the similarity among soybean treatments in Santo Antônio de Goiás during the 2022/23 season.

The dendrogram revealed the formation of four main groups among the soybean treatments. The first group, comprising treatments 8, 1, 11 and 19, exhibited similar response profiles, likely reflecting intermediate performance in terms of growth and nutrient accumulation (Figure 7).

The second group included treatments 16, 14, 20, 18 and 7, while the third group, more compact yet diverse, was composed of treatments 15, 10, 13, 4, 3 and 6. These treatments were characterized by greater GY and nutrient efficiency, consistent with the results of the PCA, especially in the case of treatments 3, 4, 15, 16 and 20. The fourth group included treatments 9, 5, 17, 2 and 12. Notably, treatment 17 was the most dissimilar within this cluster, likely due to poorer or atypical responses in one or more of the evaluated variables (Figure 7).

Across all three locations, the multivariate analysis consistently indicated that explanatory variables related to plant GY, nutrient use efficiency and root and shoot development were the main drivers of treatment differentiation. In general, PUE, GY, grains number (GN), SDM, RDM, TRL and TRSA showed the greatest contribution to the principal components. Conversely, RD and TP exhibited limited or inconsistent associations with yield‐related variables, indicating a lower overall contribution to the multivariate patterns observed across environments.

### 3.4 Grain Yield

Soybean GY showed significant differences among treatments and varied according to location. In Santo Antônio de Goiás during the 2021/22 season, treatment 2 was superior to the others, yielding 4516.4 kg ha^−1^. Treatments 1, 3, 4, 5, 6, 10, 11, 12, 13 and 14 also performed well, showing results similar to treatment 20. In Ceres during the 2021/22 season, four treatments (6, 4, 11 and 15) achieved statistically similar yields, obtaining similar results to treatments 17 and 20 and were superior to the other treatments. In Santo Antônio de Goiás during the 2022/23 season, treatments 1, 3, 4, 5, 6, 8, 9, 10, 13 and 15 contributed to the highest yields, surpassing the others but showing no significant difference compared to treatment 19 (Table 4).

**TABLE 4:** Mean grain yield values (kg ha^−1^) of soybeans grown in Santo Antônio de Goiás (2021/22 season), Ceres (2021/22 season) and Santo Antônio de Goiás (2022/23 season).

Treatments	SAG 21/22	CE 21/22	SAG 22/23
1	4.015b	1.661b	3.987a
2	4.516a	1.708b	3.710b
3	3.912b	1.731b	4.099a
4	3.805b	1.931a	4.007a
5	3.961b	1.751b	3.957a
6	3.783b	2.075a	4.088a
7	3.661c	1.687b	3.489b
8	3.507c	1.736b	3.898a
9	3.528c	1.716b	3.849a
10	3.933b	1.787b	4.049a
11	3.904b	1.970a	3.793b
12	3.787b	1.548b	3.745b
13	3.909b	1.817b	4.125a
14	3.881b	1.504b	3.607b
15	3.202d	1.944a	4.058a
16	3.601c	1.706b	3.574b
17	3.514c	2.076a	3.703b
18	3.562c	1.632b	3.709b
19	3.180d	1.764b	3.864a
20	3.945b	1.932a	3.658b
CV %	6.7	10.8	8.6

*Note:* Means followed by the same lowercase letter within a column do not differ statistically according to the Scott‐Knott test at a 5% probability level. Treatments: (1) 1381; (2) Ab‐V6; (3) 1341; (4) 1301; (5) 1301 + 1381; (6) 1301 + Ab‐V6; (7) 1301 + 1341; (8) 1254; (9) 1254 + 1381; (10) 1254 + Ab‐V6; (11) 1254 + 1341; (12) S22; (13) S22 + 1381; (14) S22 + Ab‐V6; (15) S22 + 1341; (16) TA; (17) TB; (18) T0; (19) T50; (20) T100.

## 4 Discussion

The results demonstrated that the combined use of bacterial consortia was effective in promoting soybean growth and GY, enabling a reduction in mineral fertilization. The superior performance of plant growth‐promoting rhizobacteria (PGPR) compared to mineral fertilization underscores the potential benefits of these consortia in sustainable soybean production systems. However, the effectiveness of the consortia varied by location, emphasising the specificity of microbial strains and their interactions with soil and climatic conditions. Environmental fluctuations, such as variations in temperature, humidity and solar radiation, can significantly influence microbial activity and, consequently, treatment efficacy.

Biomass accumulation reflects the plant's efficiency in capturing light, performing photosynthesis and utilising available resources (nutrients, water, space). Plants with well‐developed shoot and RDM typically exhibit enhanced physiological and reproductive performance, which directly impacts GY (Guimarães et al. 2021).

In Santo Antônio de Goiás during the 2021/22 season, SDM and RDM were strongly correlated with the positive quadrant of PC2. Treatments involving *Paenibacillus pabuli* + *Azospirillum brasilense* , *Bacillus subtilis* + *Stenotrophomonas maltophilia* and partial phosphate fertilization were closely associated with these variables, suggesting they effectively promoted biomass accumulation. The robust biomass performance of these treatments indicates enhanced photosynthetic capacity, balanced nutrient uptake and improved root development, which supported the high GYs observed.

In Ceres during the same season, the PCA revealed similar results, with SDM and RDM strongly aligned with the positive PC1 axis, which also included the production components and most root traits. The *A. brasilense* isolate and the commercial BiomaPhos treatment clustered near these vectors. Once again, the direct relationship between biomass and GY was evident, with the *A. brasilense* treatment standing out as one of the most productive.

In Santo Antônio de Goiás during the 2022/23 season, PCA results showed that SDM and RDM were associated with positive PC1, along with GY. Treatments with *Bacillus pumilus* and *Bacillus subtilis* were located near the biomass vectors, indicating greater dry matter accumulation. These treatments were among the most productive, reinforcing the strong connection between vigorous vegetative development and final yield.

The findings by Silva et al. (2020) support those of the present study, reporting increased SDM and a 25% increase in RDM when soybean plants were co‐inoculated with isolates 1301 ( *B. pumilus* ) and BRM 32114 (*Serratia* sp.). Similarly, Chagas Junior et al. (2022) observed a 52.1% increase in SDM with the use of *B. subtilis* .

Production components such as the pods number per plant (NP) and grains number per plant (GN) are directly related to soybean reproductive capacity and overall yield. The use of plant growth‐promoting microorganisms (PGPMs) can positively affect these traits by influencing plant metabolism, improving nutrient availability and stimulating reproductive development (Rezende et al. 2021).

In Santo Antônio de Goiás during the 2021/22 season, PN and GN were correlated with the negative side of PC2, along with variables such as TRSA, RD and phosphorus content in the shoot (TP). Treatments involving *S. maltophilia* , *P. pabuli* , *B. pumilus* + *B. pumilus* and *P. pabuli* + *S. maltophilia* were located near these vectors, indicating a lower pods and grains number per plant.

Conversely, in Ceres during the same season, PN and GN were associated with the positive side of PC1, along with SDM and RDM. Treatments with *P. pabuli* + *S. maltophilia* and *B. subtilis* + *S. maltophilia* were positioned near the PN and GN vectors, suggesting better reproductive performance. This confirms that pod and grain formation are closely tied to a positive inoculant response.

In Santo Antônio de Goiás during the 2022/23 season, the grains number per plant (NG) was primarily correlated with the positive PC1 axis, in alignment with GY and biomass variables, while the pods number per plant (PN) was associated with the negative PC2 axis. These results underscore the strong relationship between yield components and variables such as root system characteristics and PUE, suggesting that nutrient absorption capacity, particularly by the roots, is a key determinant of soybean yield.

These findings indicate that the combination of *P. pabuli* + *S. maltophilia* enhances the plant's efficiency in resource allocation toward pod and grain formation. Physiologically, this may be attributed to the microbial production of phytohormones such as auxins, cytokinins and gibberellins, which play essential roles in regulating flowering, pod filling and cell division (Dhouib et al. 2019). Furthermore, improved nutrient uptake, especially of nitrogen and phosphorus, promoted by PGPMs likely supported the greater development of reproductive structures and, consequently, increased final GY.

The results obtained in this study are supported by Pacentchuck et al. (2020), who reported that co‐inoculation with *Bacillus* sp. and *Azospirillum* sp. in the planting furrow increased NG. Similarly, Silva et al. (2023) found that co‐inoculation with isolates BRM 32114 and BRM 63573 significantly increased NP in soybean plants.

GY, a key indicator of agronomic success, varied among experiments, reflecting the influence of environmental conditions, nutrient management and plant–microbe interactions. The combined analysis of the three experiments, using both PCA and cluster analysis (dendrograms), enabled the identification of the most efficient treatments in promoting crop yield.

In Santo Antônio de Goiás during the 2021/22 season, GY showed a strong association with the positive PC1 axis. Treatments with *A. brasilense* , *B. pumilus* , *B. subtilis* and the commercial inoculants *Azospirillum brasilense* and BiomaPhos, along with conventional mineral fertilization, were closely associated with the GY vector in the PCA. These results suggest that both microbial consortia and complete mineral fertilization were effective in promoting biomass accumulation and efficient assimilate allocation to reproductive organs, resulting in increased yield.

A similar trend was observed in the Ceres experiment during the same season (2021/22), where the commercial inoculants *Azospirillum brasilense* and BiomaPhos, as well as conventional fertilization, were located near the GY vector in the PCA. These treatments also appeared to enhance vegetative growth, indicating a positive interaction between inoculated plants and the microbial strains.

In the 2022/23 season in Santo Antônio de Goiás, GY was most strongly explained by the PC1 axis, with the treatments *B. pumilus* , *P. pabuli* + *B. pumilus* , *B. subtilis* and *B. subtilis* + *B. pumilus* positioned closest to the GY vector in the PCA. Among these, the *B. subtilis* combination consistently stood out as a key promoter of high GY, indicating effective microbial synergism in enhancing plant growth and yield.

The repeated prominence of *B. subtilis* based treatments across both seasons in Santo Antônio de Goiás highlights their potential as promising bioinputs, even outperforming conventional fertilization strategies. These results reinforce the importance of selecting efficient and environmentally adapted microbial strains capable of acting through multiple mechanisms, including biological nitrogen fixation, phosphorus solubilization, phytohormone production and root growth stimulation. Such multifaceted action contributes to improved yield components and ultimately higher GY.

In the 2021/22 season in Santo Antônio de Goiás, the *A. brasilense* treatment alone surpassed conventional fertilization by more than 14%, equating to nearly 10 additional 60 kg bags per hectare. In Ceres during the same season, the consortia *B. pumilus* + *A. brasilense* , *P. pabuli* + *B. pumilus* , *B. subtilis* + *B. pumilus* and the *B. pumilus* isolate stood out, showing performance comparable to the commercial inoculant BiomaPhos and conventional fertilization. These treatments outperformed the commercial *Azospirillum brasilense* inoculant and partial phosphate fertilization, yielding on average nearly five more 60 kg bags than the former and four more than the latter, representing increases of approximately 16% and 12%, respectively.

It is important to highlight the efficiency of these inoculated treatments, as they delivered comparable or superior GY relative to commercial inoculants and full phosphate fertilization. This equivalence in agronomic response underscores the potential of microbial inoculants as viable, sustainable and economically attractive alternatives. In addition to sustaining high productivity, these treatments can substantially reduce production costs by lowering dependency on expensive phosphate fertilizers, representing a major advance toward environmentally friendly agriculture with improved profitability for farmers.

During the experiments, there was a slight increase in temperature ranging from 32°C to 35°C and low precipitation in Ceres during the 2021/22 season, particularly at the end of the cycle (Figure 1A–C). Despite these conditions, soybean GY in both seasons in Santo Antônio de Goiás remained above the national average of 3560 kg ha^−1^ (Companhia Nacional de Abastecimento [CONAB] 2025). In contrast, yields in Ceres were significantly below the national average, which can be attributed to the elevated temperatures, reaching up to 35°C and limited rainfall. These environmental conditions may have negatively impacted the survival and activity of beneficial microorganisms, as well as hindered their mobility in the soil. According to Gunnabo et al. (2019), environmental factors such as pH, temperature, humidity, salinity, nitrogen availability, altitude and latitude can significantly affect the symbiotic efficiency of rhizobia and the inoculation response of common bean plants.

Braga Junior et al. (2021) reported that *Bacillus subtilis* increased soybean GY by 26.6% and 15.4% in the 2015/16 and 2016/17 seasons, respectively, while also promoting plant growth through indole‐3‐acetic acid synthesis and non‐labile phosphate solubilization. Similarly, Frasca et al. (2022) found that co‐inoculation with *Serratia marcescens* (BRM 32114) and *Bacillus* sp. (BRM 63573) led to a 14.83% increase in soybean GY. In a study closely aligned with the present work, Guimarães and Klein (2023) demonstrated that an inoculant containing *B. megaterium* and *B. subtilis* , combined with half the recommended phosphorus dose, was effective in enhancing growth and nutrient uptake, resulting in higher GY compared to the control (half dose without inoculation) and comparable to full phosphorus fertilization.

Root system characteristics play a fundamental role in nutrient uptake, abiotic stress tolerance and the overall promotion of soybean growth and GY. The use of PGPMs can significantly influence parameters such as TRL, TRSA, RV and RD, enhancing the plant's ability to exploit soil resources (Guimarães et al. 2021).

In the 2021/22 season in Santo Antônio de Goiás, PCA revealed a strong correlation of root variables, particularly TRL and RV, with the positive PC1 axis. Treatments such as *B. subtilis* + *S. maltophilia* , *B. subtilis* + *B. pumilus* and partial phosphate fertilization were located near these vectors, indicating enhanced root development. This robust root architecture likely facilitated better nutrient and water uptake, contributing to improved GY, especially in the *B. subtilis* + *B. pumilus* treatment, which also performed well in other agronomic parameters.

A similar trend was observed in Ceres during the same season. Root traits such as RV, TRSA and TRL were also strongly associated with the positive PC1 axis. Treatments including *A. brasilense* , *B. pumilus* + *B. pumilus* , *P. pabuli* and *P. pabuli* + *S. maltophilia* were located near these vectors, showing promise in root system development as well as overall agronomic performance, including biomass and reproductive traits. Dendrogram analysis further supported the similarity of these treatments to other high‐performing groups, clustering them into branches associated with vigorous root development.

In the 2022/23 season in Santo Antônio de Goiás, the PCA once again revealed an association between root system traits and the PC1 axis, particularly highlighting TRSA, TRL and RV. The isolate *B. pumilus* , the commercial *Azospirillum brasilense* and conventional phosphate fertilization were positioned closest to these root‐related vectors. On the positive PC2 axis, RD was most prominent, particularly in treatments with *B. pumilus* + *A. brasilense* and *B. subtilis* + *A. brasilense* . Among these, both *B. pumilus* and the commercial *A. brasilense* treatments also stood out in GY, suggesting that a robust root system not only supported vegetative growth but also contributed to greater assimilate allocation to reproductive organs.

Although indole‐3‐acetic acid content was not directly measured in this study, the observed improvements in root development likely stem from increased microbial IAA production, which stimulates root elongation and branching, thereby enhancing nutrient uptake and yield potential in soybean. According to Rondina et al. (2020), microbial‐derived hormones like IAA can directly influence root system architecture and stimulate the formation of lateral roots, which are key to improved water and nutrient absorption.

From a physiological and microbiological standpoint, the strains evaluated likely act via the synthesis of auxins and other phytohormones that promote root elongation and branching, in addition to solubilising phosphorus, an essential nutrient for root development. The resulting increases in RV and surface area enhance interactions with soil and rhizospheric microorganisms, improving nutrient absorption efficiency and positively impacting GY (Rezende et al. 2021).

According to Lima et al. (2021), inoculation with PGPMs can significantly alter root morphology, primarily by increasing root proliferation, which translates into enhanced plant development due to improved water and nutrient uptake. Similarly, Rondina et al. (2020) demonstrated that co‐inoculation with *Bradyrhizobium* and *A. brasilense* in soybean led to increases in TRL, root length density in the soil and both the incidence and length of root hairs, ultimately resulting in increased GY.

Phosphorus (P) is a critical nutrient for soybean crops, participating in essential physiological processes such as photosynthesis, energy transfer and reproductive development. PUE is a key agronomic indicator that reflects a plant's ability to convert absorbed P into biomass and yield. The use of PGPMs can improve both phosphorus accumulation in plant tissues and PUE, especially under conditions of low soil phosphorus availability (Dhouib et al. 2019).

In the 2021/22 season in Santo Antônio de Goiás, PCA positioned PUE strongly along the positive PC1 axis. The treatment with *B. pumilus* + *A. brasilense* was located near this vector, indicating superior performance in phosphorus uptake and utilization. This treatment also showed notable results in dry matter accumulation and reproductive components, suggesting that higher phosphate efficiency contributed directly to increased crop yield.

In the Ceres experiment conducted during the same season, PCA indicated a strong positive correlation of phosphorus content (PT) with the main components, particularly PC1. Treatments with *B. pumilus* + *B. pumilus* , *P. pabuli* and *P. pabuli* + *S. maltophilia* were most closely associated with PT. These treatments exhibited not only elevated phosphorus content but also enhanced root and reproductive development, which was reflected in GY. The dendrogram clustered these treatments together, reinforcing their consistent phosphorus efficiency profiles.

In the 2022/23 season in Santo Antônio de Goiás, PUE again showed a consistent behaviour, with PCA placing it along the positive PC1 axis near the *P. pabuli* + *A. brasilense* treatment. The efficiency of this treatment may be attributed to microbial mechanisms such as the solubilization of inorganic phosphates, mineralization of organic phosphorus compounds and stimulation of root growth, which expands the soil exploration zone (Rawat et al. 2021; Raymond et al. 2020).

Overall, the treatments that most positively influenced GY across experiments included *A. brasilense* , *B. subtilis* , commercial *A. brasilense* , BiomaPhos and conventional phosphate fertilization. These findings confirm the critical role of PUE as a key determinant of productivity. This efficiency can be ascribed to synergistic interactions between microbial strains and the root system, as well as biochemical processes in the rhizosphere that enhance phosphorus availability to plants.

Considering that the optimal phosphorus content for soybean ranges from 2.5 to 5.0 g kg^−1^ (Bargaz et al. 2021), the results across all experiments were satisfactory, with average values exceeding the minimum threshold of 2.5 g kg^−1^. Despite high TP stocks in the experimental soils, less than 0.1% is readily available for plant uptake (Filiz et al. 2022). Moreover, the efficient absorption and translocation of phosphorus require favourable environmental conditions, including soil temperatures around 30°C and adequate moisture (Rengel et al. 2022). Under the environmental conditions present in this study, consortia containing *Bacillus* strains proved effective in hydrolysing both organic and inorganic phosphorus compounds, thereby improving P availability.

Batista et al. (2018) highlighted *B. cereus* and *B. megaterium* as promising phosphate‐solubilising rhizobacteria due to their multifunctional traits conducive to increased phosphorus bioavailability. Similarly, Izydorczyk et al. (2022) reported that applications of *B. cereus* and *B. thuringiensis* positively influence plant growth by solubilising phosphate deposits that are otherwise insoluble and inaccessible to plants.

Overall, the results indicate that the performance of multifunctional inoculants is strongly dependent on local edaphoclimatic conditions. In Santo Antônio de Goiás, treatments based on *Azospirillum brasilense* , *Bacillus subtilis* and *Bacillus pumilus* consistently showed superior associations with biomass accumulation, PUE and GY, performing similarly or better than conventional fertilization. In Ceres, where higher temperatures and lower rainfall prevailed, consortia containing *Bacillus* spp. stood out, suggesting greater microbial adaptability and stability under more restrictive environmental conditions. These findings demonstrate that the effectiveness of multifunctional inoculants is environment‐specific and highlight the importance of selecting microbial consortia according to geographic and climatic conditions to maximize soybean GY and sustainability.

Beyond agronomic performance, this study advances the understanding of PGPMs by demonstrating that microbial functions and effectiveness are strongly shaped by plant–microbe–environment interactions. The differential responses of bacterial taxa and consortia reveal that functions such as biomass accumulation, root system modulation and PUE are expressed in a context‐dependent manner, influenced by edaphoclimatic conditions. These findings reinforce the view of microbial inoculants as dynamic biological agents whose ecological adaptability and functional compatibility with local environments are key determinants of their agronomic success.

## 5 Conclusion

The combination of BRM 67207 ( *Bacillus subtilis* ) + BRM 67206 ( *Bacillus pumilus* ) emerged as the most promising treatment for soybean cultivation, standing out repeatedly between harvests, indicating high environmental adaptability and multifunctional performance. This co‐inoculation consistently promoted significant gains in shoot and RDM, pods number per plant, root length, diameter and volume, as well as PUE and, most notably, GY.

This study is limited by its focus on specific regions of Central Brazil and a restricted evaluation period, which may constrain the broader applicability of the results. Further multi‐site and multi‐year assessments are needed to validate the performance of these microbial consortia under diverse soil and climatic conditions. Future investigations should also explore long‐term effects on soil health and the integration of these inoculants into broader sustainable management strategies.

## Author Contributions

**Cássia Cristina Rezende Mirza:** conceptualization, data curation, formal analysis, investigation, methodology, visualization, writing – original draft, writing – review and editing. **Ana Paula Santos Oliveira:** conceptualization, formal analysis, methodology, visualization, writing – review and editing. **Maria Eduarda Silvério Mateus:** conceptualization, visualization, methodology. **Princewill Chukwuma Asobia:** visualization, methodology, conceptualization. **Cleiton Mateus Sousa:** formal analysis, methodology, visualization, writing – review and editing. **Laylla Luanna de Mello Frasca:** conceptualization, methodology, visualization. **Mariana Aguiar Silva:** methodology, visualization, conceptualization. **Enderson Petrônio de Brito Ferreira:** funding acquisition, formal analysis, investigation, supervision, validation, writing – review and editing, visualization.

## Funding

This work was supported by Empresa Brasileira de Pesquisa Agropecuária, 20.20.00.061.00.00; INCT‐Plant Growth Promoting Microorganisms for Agricultural Sustainability and Environmental Responsibility, CNPq 465133/2014‐4; Fundação Araucária, STI 043/2019; Conselho Nacional de Desenvolvimento Científico e Tecnológico, 313827/2020‐6.

## Ethics Statement

The authors used OpenAI's ChatGPT (GPT‐5 model, 2026) in a limited manner during the preparation of this manuscript. The AI tool was used exclusively to assist with English language refinement, grammar correction, sentence clarity and organization of the text flow in selected sections of the manuscript, including the Introduction, Discussion and Conclusion. All prompts, interpretations, scientific analyses and final decisions regarding the content were performed and critically reviewed by the authors. The authors take full responsibility for the accuracy, originality and integrity of the manuscript content.

## Conflicts of Interest

The authors declare no conflicts of interest.

## Data Availability

The data that support the findings of this study are available on request from the corresponding author. The data are not publicly available due to privacy or ethical restrictions.
